# Zona chez le nourrisson: à propos d’un cas

**Published:** 2010-10-31

**Authors:** Kaoutar Zinelabidine, Meriame Meziane, Oufae Mikou, Fatima Zahra Mernissi

**Affiliations:** 1 Service de Dermatologie, CHU Hassan II, Fès, Maroc

**Keywords:** nourrisson, antiviraux, zona, varicelle

## Abstract

Le zona est dû à la réactivation du virus varicelle-zona, celui-ci gagne les ganglions sensitif à partir d’un foyer cutané ou muqueux lors de la primo infection. La survenue de zona chez le nourrisson est une situation rare, celle-ci se caractérise par la prédominance des signes fonctionnelles à type de fiàvre et de céphalée est l’absence de douleur post zostérienne. Le diagnostic est clinique et doit être évoqué devant des lésions vésiculeuses groupés en bouquet et dont la disposition suit un métamàre. Les complications chez le nourrisson sont rares. Le traitement fait appel aux antiviraux.

## Introduction

Le zona du nourrisson est une situation rare, due le plus souvent et non exclusivement à une exposition intra utérine au virus varicelle–zona (VZV), il constitue une forme bénigne de zona, marqué surtout par la présence des signes généraux et l'absence de douleur. Le traitement fait appel aux antiviraux, les complications sont rares à type de surinfection, de dépigmentation et d'ulcération.

## Observation

**Figure 1:**
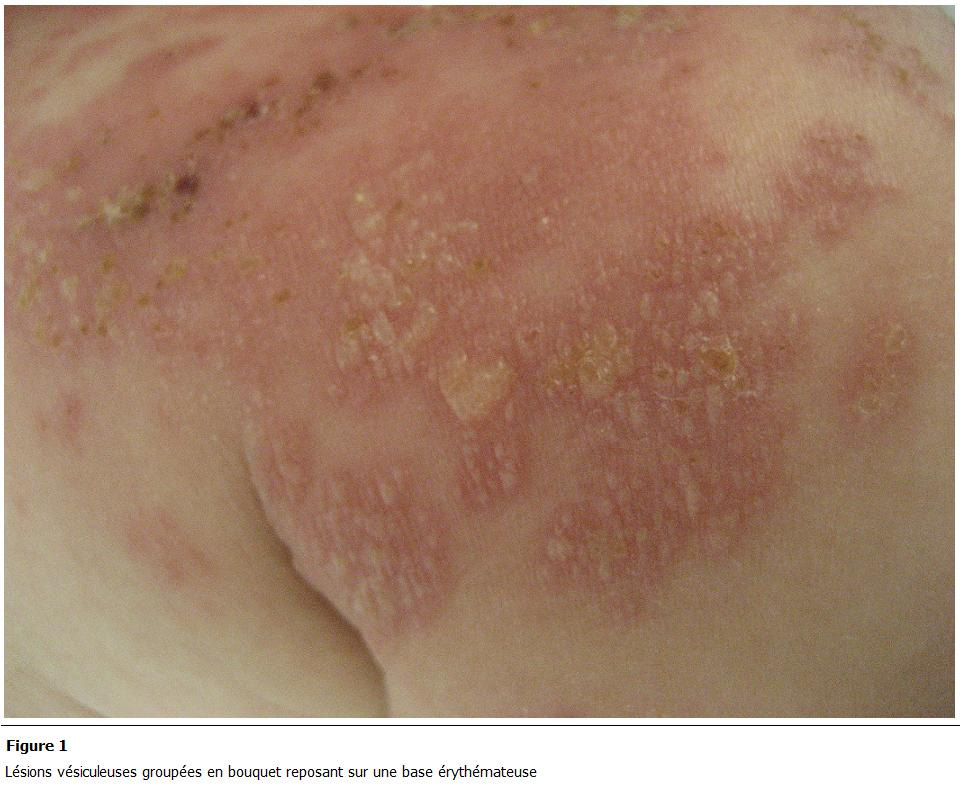
Lésions vésiculeuses groupées en bouquet reposant sur une base érythémateuse

**Figure 2:**
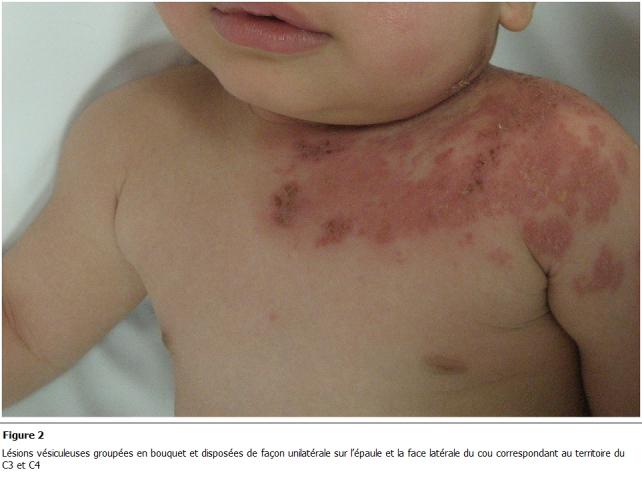
lésions vésiculeuses groupées en bouquet et disposées de façon unilatérale sur l’épaule et la face latérale du cou correspondant au territoire du C3 et C4

Le consentement écrit des gardiens de l’enfant a été obtenu pour la présentation de ce cas clinique. Nourrisson A, abandonné, âgé de 11 mois, sans antécédents pathologiques notables qui présentait 2 jours avant la consultation des lésions vésiculeuses au niveau de l’épaule gauche. évoluant dans un contexte fébrile. L’examen clinique trouvait un nourrisson calme, en bon état général, fébrile à 38°, l’examen dermatologique objectivait des lésions vésiculeuses groupées en bouquet reposant sur une base érythémateuse (Figure 1) de façon unilatérale sur l’épaule et la face latérale du cou correspondant au territoire du C3 et C4 (Figure 2). Le diagnostic de zona a été retenu sur l’aspect clinique des lésions. Le reste de l'examen n’objectivait pas de signes neurologiques ni d'adénopathies ni d'autres signes. Une numération formule sanguine a été réalisée et n'objectivait pas d’anomalie. Notre patient a été mis sous aciclovir à raison de 20 mg/kg 4 fois/j en association avec un traitement symptomatique à base d’antiseptique et d'antipyrétique avec une bonne évolution au bout de 5 jours.

## Discussion

Le virus varicelle-zona (VZV) appartient à la famille des Herpes viridae, c'est un virus à enveloppe, avec génome à ADN, l'homme est le réservoir exclusif du virus. Il a une affinité particulière pour la peau, le système nerveux et les poumons.

La majorité des cas de zona de l’enfant surviennent après l’âge de 5ans, Parmi tous les cas rapportés de zona, moins de 10% ont moins de 20 ans, et 5% ont moins de 15 ans [1], et une incidence de 0,74 pour 1000 chez la population de moins de 9 ans [2]. 26 cas de zona du nourrisson ont été rapportés dans la littérature. Parmi ces cas, 15 semblent issus d’une exposition intra-utérine et 6 cas n’avaient aucune histoire d’exposition antérieure au virus [3,4], notre patient était abandonné ainsi on n'avait pas de renseignements sur les antécédents de la maman. L'incidence du zona est plus accentuée chez les nourrissons ayant une hémopathie [2], notre nourrisson n'avait pas d'adénopathies et la numération de formule sanguine était normale.

Apres la primo-infection, le virus gagne les ganglions sensitifs par voie hématogène et/ou neurogène à partir de la peau et les muqueuses, lors de la réactivation, il migre le long des fibres nerveuses sensitives jusqu'à la peau. Une éruption vésiculeuse caractéristique apparait alors dans le métamère correspondant au ganglion rachidien colonisé lors de la primo infection. Sa survenue chez le nourrisson est probablement liée à une réponse immunitaire immature à cette l’infection. Les taux bas de lymphocytes, des cytokines et des immunoglobulines spécifiques, pourraient avoir pour conséquence l’incapacité de maintenir la latence de VZV et aboutir à l’apparition d’un zona à cet âge. Le diagnostic est clinique devant de lésions vésiculeuses groupées en bouquet et dont la disposition suit un métamère, les localisations cervicale et sacrée sont les plus fréquentes chez le nourrisson [5-7]. Les examens biologiques ne sont pas obligatoires. Le cytodiagnostic de Tzanck, effectué par grattage de la base de la lésion, peut montrer des cellules géantes, et constitue un argument pour la confirmation diagnostic [6].

Le zona du nourrisson ne s’accompagne pas habituellement de douleur ou de névralgie post zostérienne. Cependant les signes généraux sont plus marqués à type de fièvre, des céphalées et une lymphadénopathie régionale. La prise en charge fait appel d'abord aux bains quotidiens, des soins locaux par un antiseptique, L’aciclovir par voie orale constitue le traitement de 1 ère intension à raison de 20 mg/Kg 4 fois /j pendant 5 à 7 jours. Les autres antiviraux tels le famciclovir et le valacyclovir ne sont pas indiqués chez le nourrisson [5].

Les différentes études s’accordent sur la bénignité de cette affection chez le nourrisson et sur un pronostic excellent, Les complications les plus fréquentes sont une surinfection bactérienne secondaire, une dépigmentation et des cicatrices, d'autres complications sont plus rares tels une encéphalite, une ventriculite, une sclérokeratite et une uvéite antérieure [5]. Les récidives sont possibles et répondent en général au traitement antiviral conventionnel.

## Conclusion

Le zona du nourrisson doit être évoqué devant une symptomatologie évocatrice, même en absence de notion de varicelle maternelle pendant la grossesse ou en période postnatale. Les signes fonctionnels sont au premier plan tel la fièvre et les céphalées, Les complications demeurent rares.

## Conflits d’intérêt

Les auteurs déclarent n’avoir aucuns conflits d’intérêts

## Contribution des auteurs

Kaoutar zinelabidine a rédigé l’article, les autres auteurs ont contribué à la prise en charge thérapeutique du nourrisson et à la rédaction de ce document.
